# Ethyl 6-(6-meth­oxy­naphthalen-2-yl)-4-(naphthalen-2-yl)-2-oxocyclo­hex-3-ene-1-carboxyl­ate

**DOI:** 10.1107/S1600536813002857

**Published:** 2013-02-06

**Authors:** Manpreet Kaur, Jerry P. Jasinski, Amanda C. Keeley, H. S. Yathirajan, Anil N. Mayekar

**Affiliations:** a Department of Studies in Chemistry, University of Mysore, Manasagangotri, Mysore 570 006, India; b Department of Chemistry, Keene State College, 229 Main Street, Keene, NH 03435-2001, USA; c SeQuent Scientific Limited, Biakampady, Mangalore 575 011, India

## Abstract

The title compound, C_30_H_26_O_4_, contains an oxo-cyclo­hexane ring in a distorted half-chair configuration, with disorder of two C atoms in a 0.859 (4):0.141 (4) ratio. The dihedral angle between the mean planes of the two napthalene ring systems is 58.6 (8)°.

## Related literature

For the biological activity of chalcones, see: Dimmock *et al.* (1999 ▶); Mayekar *et al.* (2010 ▶). For their synthesis, see: Dhar (1981 ▶). For related structures, see: Harrison *et al.* (2010 ▶); Li *et al.* (2009 ▶); Kaur *et al.* (2012) ▶. For standard bond lengths, see Allen *et al.* (1987 ▶).
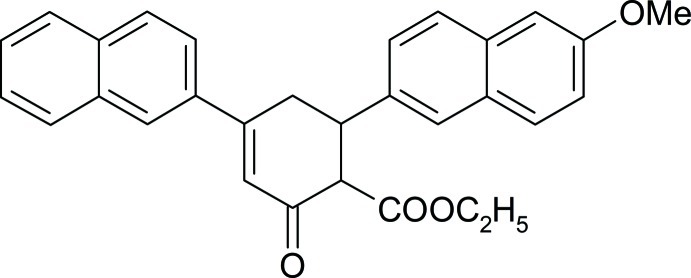


## Experimental

### 

#### Crystal data

C_30_H_26_O_4_
*M* *_r_* = 450.51
Monoclinic, 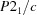

*a* = 18.4688 (10) Å
*b* = 11.2940 (6) Å
*c* = 10.9676 (5) Å
β = 96.082 (5)°
*V* = 2274.8 (2) Å^3^
*Z* = 4
Cu *K*α radiation
μ = 0.69 mm^−1^
*T* = 173 K
0.24 × 0.18 × 0.06 mm

#### Data collection

Agilent Xcalibur (Eos, Gemini) diffractometer
Absorption correction: multi-scan (*CrysAlis PRO* and *CrysAlis RED*; Agilent, 2012 ▶) *T*_min_ = 0.864, *T*_max_ = 1.000
14336 measured reflections
4461 independent reflections
3497 reflections with *I* > 2σ(*I*)
*R*_int_ = 0.037

#### Refinement

*R*[*F* ^2^ > 2σ(*F* ^2^)] = 0.055
*wR*(*F* ^2^) = 0.166
*S* = 1.09
4461 reflections
317 parameters
H-atom parameters constrained
Δρ_max_ = 0.30 e Å^−3^
Δρ_min_ = −0.22 e Å^−3^

### 

Data collection: *CrysAlis PRO* (Agilent, 2012 ▶); cell refinement: *CrysAlis PRO*; data reduction: *CrysAlis RED* (Agilent, 2012 ▶); program(s) used to solve structure: *SHELXS97* (Sheldrick, 2008 ▶); program(s) used to refine structure: *SHELXL97* (Sheldrick, 2008 ▶); molecular graphics: *SHELXTL* (Sheldrick, 2008 ▶); software used to prepare material for publication: *SHELXTL*.

## Supplementary Material

Crystal structure: contains datablock(s) global, I. DOI: 10.1107/S1600536813002857/fj2612sup1.cif

Structure factors: contains datablock(s) I. DOI: 10.1107/S1600536813002857/fj2612Isup2.hkl

Supplementary material file. DOI: 10.1107/S1600536813002857/fj2612Isup3.cml

Additional supplementary materials:  crystallographic information; 3D view; checkCIF report

## Figures and Tables

**Fig. 1.**
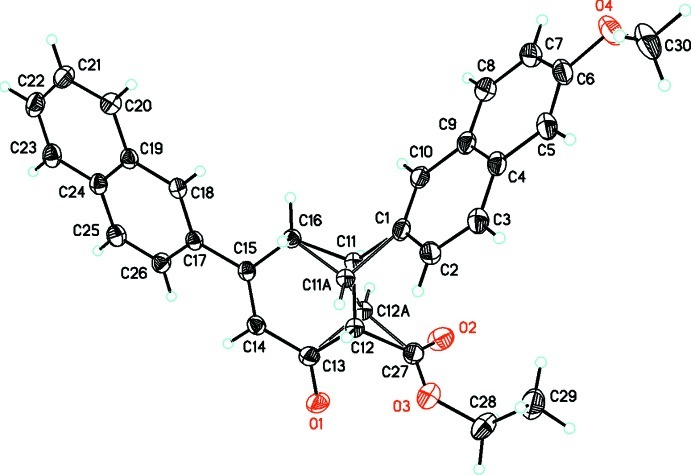
Molecular structure of the title compound showing the atom labeling scheme and 50% probability displacement ellipsoids.

**Fig. 2.**
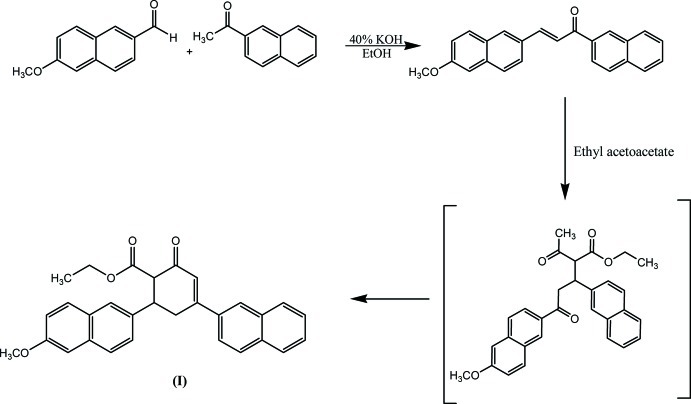
Synthesis of the title compound, (I).

## supplementary crystallographic information

### Comment

Chalcones and their corresponding heterocyclic analogs are valuable intermediates in organic synthesis (Dhar, 1981). This scaffold is found in various medicinally useful compounds and is known to exhibit a multitude of biological activities (Dimmock *et al.*, 1999). The crystal structures of (±)-ethyl 6-(6-methoxy-2-naphthyl)-4- (4-methylphenyl)-2-oxocyclohex-3-ene-1-carboxylate (Li *et al.*, 2009) and ethyl 4-(2,4-dichlorophenyl)-6-(6-methoxy-2- naphthyl)-2-oxocyclohex-3-ene-1-carboxylate (Harrison *et al.*, 2010) have been reported. Recently, we have reported the crystal and molecular structure studies of ethyl 4-(4-hydroxyphenyl)-6-(6-methoxy- 2-naphthyl)-2-oxocyclohex-3-ene-1-carboxylate and ethyl 4-(3- bromophenyl)-6-(6-methoxy-2-naphthyl)-2-oxocyclohex-3-ene-1-carboxylate (Manpreet Kaur *et al.*, 2012). As a part of our ongoing structural studies of substituted cyclohexene carboxylates, this paper reports the crystal structure of the title compound, (I), C_30_H_26_O_4_.

In the title compound the asymmetric unit consists of an ortho bonded ethyl carboxylate group and meta bonded naphthyl and methoxy-naphthyl groups to a disordered oxo-cyclohexane ring with C11 and C12 in a 0.859 (4):0.141 (4) ratio. The dihedral angle between the mean planes of the two napthalene ring systems is 58.6 (8)°. The napthalene and methoxynapthalene rings are twisted by 79.5 (7)° and 72.5 (9)° from the mean plane of the carboxylate group. Bond lengths are in normal ranges (Allen *et al.*, 1987).

### Experimental

The title compound was synthesized as reported earlier (Mayekar *et al.*, 2010).

Preparation of (2E)-3-(6-methoxy-2-naphthyl)-1-(1-naphthyl)prop-2-en -1-one. To a thoroughly stirred solution of 6-methoxy-2-naphthaldehyde (1.86 g, 10 mmol) and 1-naphthalen-2-yl-ethanone (1.70 g, 10 mmol) in 15 ml methanol, 5 ml of 40% KOH solution was added. The reaction mixture was stirred overnight and the solid separated was collected by filteration. The product obtained was recrystallized from methanol.

Preparation of ethyl 4-(1-naphthyl)-6-(6-methoxy-2-naphthyl)-2- oxocyclohex-3-ene-1-carboxylate 3-(6-Methoxy-naphthalen-2-yl)-1- naphthalen-2-yl-propenone. (1.69 g, 5 mmol) and ethyl acetoacetate (5 mmol) were refluxed for 4-6 hrs in 15 ml ethanol in presence of 0.8 ml of 10% NaOH. The reaction mixture was cooled to room temperature and the reaction mass was filtered. The compound (I) was recrystallized from methanol (Fig 2).

The compound was further recrystallized from a 1:1 mixture of toluene & dimethylformamide by slow evaporation (m.p.: 440-443 K).

### Refinement

All the H atoms were placed in their calculated positions and then refined using the riding model with Atom—H lengths of 0.93Å (CH), 0.97Å (CH_2_) or 0.96Å (CH_3_). Isotropic displacement parameters for these atoms were set to 1.18-1.21 (CH, CH_2_) or 1.49 (CH_3_) times *U*_eq_ of the parent atom.

### Crystal data

**Table:**

C_30_H_26_O_4_	*F*(000) = 952
*M**_r_* = 450.51	*D*_x_ = 1.315 Mg m^−^^3^
Monoclinic, *P*2_1_/*c*	Cu *K*α radiation, λ = 1.54184 Å
Hall symbol: -P 2ybc	Cell parameters from 4292 reflections
*a* = 18.4688 (10) Å	θ = 3.9–72.7°
*b* = 11.2940 (6) Å	µ = 0.69 mm^−^^1^
*c* = 10.9676 (5) Å	*T* = 173 K
β = 96.082 (5)°	Chunk, colorless
*V* = 2274.8 (2) Å^3^	0.24 × 0.18 × 0.06 mm
*Z* = 4	

### Data collection

**Table:**

Agilent Xcalibur (Eos, Gemini) diffractometer	4461 independent reflections
Radiation source: Enhance (Cu) X-ray Source	3497 reflections with *I* > 2σ(*I*)
Graphite monochromator	*R*_int_ = 0.037
Detector resolution: 16.0416 pixels mm^-1^	θ_max_ = 72.9°, θ_min_ = 4.6°
ω scans	*h* = −22→22
Absorption correction: multi-scan (*CrysAlis PRO* and *CrysAlis RED*; Agilent, 2012)	*k* = −11→13
*T*_min_ = 0.864, *T*_max_ = 1.000	*l* = −13→13
14336 measured reflections	

### Refinement

**Table:**

Refinement on *F*^2^	Primary atom site location: structure-invariant direct methods
Least-squares matrix: full	Secondary atom site location: difference Fourier map
*R*[*F*^2^ > 2σ(*F*^2^)] = 0.055	Hydrogen site location: inferred from neighbouring sites
*wR*(*F*^2^) = 0.166	H-atom parameters constrained
*S* = 1.09	*w* = 1/[σ^2^(*F*_o_^2^) + (0.0709*P*)^2^ + 1.1847*P*] where *P* = (*F*_o_^2^ + 2*F*_c_^2^)/3
4461 reflections	(Δ/σ)_max_ < 0.001
317 parameters	Δρ_max_ = 0.30 e Å^−^^3^
0 restraints	Δρ_min_ = −0.22 e Å^−^^3^

### Special details

**Table:**

Geometry. All esds (except the esd in the dihedral angle between two l.s. planes) are estimated using the full covariance matrix. The cell esds are taken into account individually in the estimation of esds in distances, angles and torsion angles; correlations between esds in cell parameters are only used when they are defined by crystal symmetry. An approximate (isotropic) treatment of cell esds is used for estimating esds involving l.s. planes.
Refinement. Refinement of *F*^2^ against ALL reflections. The weighted *R*-factor *wR* and goodness of fit *S* are based on *F*^2^, conventional *R*-factors *R* are based on *F*, with *F* set to zero for negative *F*^2^. The threshold expression of *F*^2^ > σ(*F*^2^) is used only for calculating *R*-factors(gt) *etc*. and is not relevant to the choice of reflections for refinement. *R*-factors based on *F*^2^ are statistically about twice as large as those based on *F*, and *R*- factors based on ALL data will be even larger.

### Fractional atomic coordinates and isotropic or equivalent isotropic displacement parameters (Å^2^)

**Table:**

	*x*	*y*	*z*	*U*_iso_*/*U*_eq_	Occ. (<1)
O1	0.38758 (10)	0.25380 (17)	1.19112 (16)	0.0537 (5)	
O2	0.22028 (11)	0.26879 (18)	1.06300 (17)	0.0604 (5)	
O3	0.24685 (10)	0.40261 (17)	1.21035 (17)	0.0536 (5)	
O4	−0.01861 (10)	0.74357 (18)	0.5654 (2)	0.0636 (5)	
C1	0.25133 (11)	0.4898 (2)	0.8777 (2)	0.0399 (5)	
C2	0.22175 (12)	0.5763 (2)	0.9533 (2)	0.0436 (5)	
H2	0.2429	0.5873	1.0332	0.052*	
C3	0.16246 (12)	0.6437 (2)	0.9100 (2)	0.0434 (5)	
H3	0.1438	0.6990	0.9612	0.052*	
C4	0.12995 (11)	0.6298 (2)	0.7896 (2)	0.0388 (5)	
C5	0.06928 (12)	0.7000 (2)	0.7404 (2)	0.0450 (6)	
H5	0.0502	0.7575	0.7888	0.054*	
C6	0.03949 (12)	0.6828 (2)	0.6232 (2)	0.0474 (6)	
C7	0.06710 (13)	0.5948 (2)	0.5493 (2)	0.0498 (6)	
H7	0.0458	0.5837	0.4694	0.060*	
C8	0.12468 (13)	0.5258 (2)	0.5935 (2)	0.0462 (6)	
H8	0.1417	0.4673	0.5441	0.055*	
C9	0.15855 (12)	0.5428 (2)	0.7140 (2)	0.0393 (5)	
C10	0.21957 (12)	0.4748 (2)	0.7618 (2)	0.0406 (5)	
H10	0.2383	0.4183	0.7120	0.049*	
C11	0.31578 (12)	0.4105 (2)	0.9216 (2)	0.0292 (6)	0.854 (6)
H11	0.3062	0.3315	0.8867	0.035*	0.854 (6)
C11A	0.3278 (8)	0.4586 (15)	0.9685 (16)	0.0292 (6)	0.146 (6)
H11A	0.3382	0.5145	1.0364	0.035*	0.146 (6)
C12	0.32545 (13)	0.3985 (2)	1.0610 (2)	0.0319 (6)	0.859 (4)
H12	0.3353	0.4767	1.0976	0.038*	0.859 (4)
C12A	0.3134 (8)	0.3335 (14)	1.0095 (14)	0.0319 (6)	0.141 (4)
H12A	0.3022	0.2738	0.9454	0.038*	0.141 (4)
C13	0.38903 (12)	0.3163 (2)	1.1010 (2)	0.0405 (5)	
C14	0.45116 (11)	0.31945 (19)	1.03020 (19)	0.0338 (5)	
H14	0.4926	0.2771	1.0592	0.041*	
C15	0.45197 (10)	0.38021 (17)	0.92505 (18)	0.0297 (4)	
C16	0.38731 (10)	0.45588 (18)	0.87838 (18)	0.0307 (4)	
H16A	0.3827	0.4568	0.7895	0.037*	
H16B	0.3958	0.5365	0.9068	0.037*	
C17	0.51453 (11)	0.37341 (17)	0.85114 (18)	0.0307 (4)	
C18	0.52236 (11)	0.45096 (18)	0.75709 (19)	0.0321 (4)	
H18	0.4881	0.5108	0.7409	0.039*	
C19	0.58099 (11)	0.44294 (19)	0.68371 (19)	0.0341 (5)	
C20	0.58938 (13)	0.5230 (2)	0.5872 (2)	0.0429 (5)	
H20	0.5557	0.5836	0.5704	0.052*	
C21	0.64671 (14)	0.5122 (2)	0.5182 (2)	0.0492 (6)	
H21	0.6516	0.5652	0.4547	0.059*	
C22	0.69812 (13)	0.4214 (2)	0.5431 (2)	0.0489 (6)	
H22	0.7368	0.4147	0.4958	0.059*	
C23	0.69187 (12)	0.3432 (2)	0.6354 (2)	0.0435 (5)	
H23	0.7265	0.2838	0.6510	0.052*	
C24	0.63308 (11)	0.35112 (19)	0.7081 (2)	0.0355 (5)	
C25	0.62426 (12)	0.2717 (2)	0.8043 (2)	0.0402 (5)	
H25	0.6578	0.2109	0.8209	0.048*	
C26	0.56774 (11)	0.28198 (19)	0.8737 (2)	0.0368 (5)	
H26	0.5636	0.2284	0.9370	0.044*	
C27	0.25774 (13)	0.3470 (2)	1.1088 (2)	0.0439 (5)	
C28	0.18501 (16)	0.3692 (3)	1.2711 (3)	0.0612 (7)	
H28A	0.1985	0.3663	1.3589	0.073*	
H28B	0.1686	0.2910	1.2441	0.073*	
C29	0.12518 (16)	0.4561 (3)	1.2428 (3)	0.0714 (9)	
H29A	0.1430	0.5345	1.2622	0.107*	
H29B	0.0860	0.4382	1.2907	0.107*	
H29C	0.1079	0.4518	1.1572	0.107*	
C30	−0.04727 (15)	0.8396 (3)	0.6307 (3)	0.0724 (9)	
H30A	−0.0116	0.9015	0.6423	0.109*	
H30B	−0.0590	0.8121	0.7091	0.109*	
H30C	−0.0904	0.8696	0.5845	0.109*	

### Atomic displacement parameters (Å^2^)

**Table:**

	*U*^11^	*U*^22^	*U*^33^	*U*^12^	*U*^13^	*U*^23^
O1	0.0508 (10)	0.0660 (12)	0.0460 (10)	0.0170 (9)	0.0129 (8)	0.0244 (8)
O2	0.0681 (13)	0.0659 (12)	0.0492 (11)	−0.0046 (10)	0.0151 (9)	−0.0009 (9)
O3	0.0495 (10)	0.0557 (10)	0.0579 (11)	−0.0018 (8)	0.0164 (8)	0.0014 (9)
O4	0.0415 (10)	0.0702 (13)	0.0753 (13)	0.0104 (9)	−0.0112 (9)	0.0109 (10)
C1	0.0290 (10)	0.0431 (12)	0.0496 (13)	0.0025 (9)	0.0130 (9)	0.0137 (10)
C2	0.0349 (11)	0.0487 (13)	0.0469 (13)	−0.0001 (10)	0.0035 (10)	0.0096 (11)
C3	0.0353 (11)	0.0427 (12)	0.0529 (14)	0.0026 (10)	0.0074 (10)	0.0022 (11)
C4	0.0287 (10)	0.0374 (11)	0.0509 (13)	−0.0027 (9)	0.0072 (9)	0.0051 (10)
C5	0.0317 (11)	0.0421 (12)	0.0609 (15)	0.0025 (9)	0.0037 (10)	0.0039 (11)
C6	0.0294 (11)	0.0495 (14)	0.0622 (16)	−0.0011 (10)	−0.0006 (10)	0.0119 (12)
C7	0.0384 (12)	0.0576 (15)	0.0527 (14)	−0.0078 (11)	0.0013 (10)	0.0062 (12)
C8	0.0400 (12)	0.0494 (14)	0.0497 (14)	−0.0042 (10)	0.0077 (10)	0.0027 (11)
C9	0.0316 (11)	0.0385 (12)	0.0493 (13)	−0.0029 (9)	0.0107 (9)	0.0064 (10)
C10	0.0340 (11)	0.0431 (12)	0.0465 (13)	0.0037 (9)	0.0125 (9)	0.0092 (10)
C11	0.0285 (11)	0.0313 (14)	0.0274 (13)	−0.0010 (10)	0.0024 (9)	−0.0005 (11)
C11A	0.0285 (11)	0.0313 (14)	0.0274 (13)	−0.0010 (10)	0.0024 (9)	−0.0005 (11)
C12	0.0305 (12)	0.0331 (13)	0.0322 (13)	0.0013 (10)	0.0046 (9)	−0.0010 (10)
C12A	0.0305 (12)	0.0331 (13)	0.0322 (13)	0.0013 (10)	0.0046 (9)	−0.0010 (10)
C13	0.0385 (12)	0.0473 (13)	0.0359 (11)	0.0068 (10)	0.0043 (9)	0.0053 (10)
C14	0.0297 (10)	0.0368 (11)	0.0345 (11)	0.0057 (8)	0.0011 (8)	−0.0018 (9)
C15	0.0281 (10)	0.0287 (10)	0.0321 (10)	−0.0016 (8)	0.0019 (8)	−0.0046 (8)
C16	0.0294 (10)	0.0311 (10)	0.0322 (10)	0.0015 (8)	0.0054 (8)	0.0013 (8)
C17	0.0274 (9)	0.0311 (10)	0.0333 (10)	−0.0017 (8)	0.0014 (8)	−0.0047 (8)
C18	0.0269 (10)	0.0333 (10)	0.0358 (11)	0.0003 (8)	0.0015 (8)	−0.0037 (8)
C19	0.0300 (10)	0.0360 (11)	0.0359 (11)	−0.0047 (8)	0.0012 (8)	−0.0048 (9)
C20	0.0409 (12)	0.0456 (13)	0.0425 (12)	−0.0024 (10)	0.0053 (10)	0.0020 (10)
C21	0.0467 (14)	0.0593 (15)	0.0430 (13)	−0.0111 (12)	0.0115 (10)	0.0027 (11)
C22	0.0393 (13)	0.0626 (16)	0.0473 (14)	−0.0107 (11)	0.0166 (10)	−0.0136 (12)
C23	0.0347 (11)	0.0484 (13)	0.0483 (13)	−0.0008 (10)	0.0080 (10)	−0.0130 (11)
C24	0.0301 (10)	0.0381 (11)	0.0383 (11)	−0.0014 (8)	0.0039 (8)	−0.0093 (9)
C25	0.0345 (11)	0.0375 (11)	0.0487 (13)	0.0077 (9)	0.0043 (9)	−0.0028 (10)
C26	0.0344 (11)	0.0360 (11)	0.0404 (12)	0.0034 (9)	0.0061 (9)	0.0024 (9)
C27	0.0367 (12)	0.0492 (14)	0.0461 (13)	0.0045 (10)	0.0066 (10)	0.0138 (11)
C28	0.0632 (17)	0.0641 (17)	0.0617 (17)	0.0050 (14)	0.0318 (14)	0.0025 (14)
C29	0.0508 (16)	0.086 (2)	0.080 (2)	0.0083 (16)	0.0212 (15)	0.0115 (18)
C30	0.0447 (15)	0.0635 (18)	0.105 (3)	0.0166 (14)	−0.0095 (16)	0.0083 (18)

### Geometric parameters (Å, º)

**Table:**

O1—C13	1.217 (3)	C12A—H12A	0.9800
O2—C27	1.199 (3)	C13—C14	1.452 (3)
O3—C27	1.312 (3)	C14—C15	1.343 (3)
O3—C28	1.432 (3)	C14—H14	0.9300
O4—C6	1.372 (3)	C15—C17	1.482 (3)
O4—C30	1.432 (4)	C15—C16	1.513 (3)
C1—C10	1.354 (3)	C16—H16A	0.9700
C1—C2	1.427 (3)	C16—H16B	0.9700
C1—C11	1.526 (3)	C17—C18	1.372 (3)
C1—C11A	1.676 (15)	C17—C26	1.429 (3)
C2—C3	1.376 (3)	C18—C19	1.419 (3)
C2—H2	0.9300	C18—H18	0.9300
C3—C4	1.400 (3)	C19—C20	1.413 (3)
C3—H3	0.9300	C19—C24	1.421 (3)
C4—C9	1.424 (3)	C20—C21	1.371 (3)
C4—C5	1.431 (3)	C20—H20	0.9300
C5—C6	1.358 (4)	C21—C22	1.405 (4)
C5—H5	0.9300	C21—H21	0.9300
C6—C7	1.412 (4)	C22—C23	1.358 (4)
C7—C8	1.365 (4)	C22—H22	0.9300
C7—H7	0.9300	C23—C24	1.417 (3)
C8—C9	1.414 (3)	C23—H23	0.9300
C8—H8	0.9300	C24—C25	1.407 (3)
C9—C10	1.417 (3)	C25—C26	1.360 (3)
C10—H10	0.9300	C25—H25	0.9300
C11—C12	1.526 (3)	C26—H26	0.9300
C11—C16	1.538 (3)	C28—C29	1.486 (4)
C11—H11	0.9800	C28—H28A	0.9700
C11A—C12A	1.51 (2)	C28—H28B	0.9700
C11A—C16	1.555 (14)	C29—H29A	0.9600
C11A—H11A	0.9800	C29—H29B	0.9600
C12—C27	1.522 (3)	C29—H29C	0.9600
C12—C13	1.525 (3)	C30—H30A	0.9600
C12—H12	0.9800	C30—H30B	0.9600
C12A—C27	1.582 (15)	C30—H30C	0.9600
C12A—C13	1.643 (15)		
			
C27—O3—C28	118.0 (2)	C14—C15—C17	121.55 (18)
C6—O4—C30	117.4 (2)	C14—C15—C16	119.93 (18)
C10—C1—C2	118.5 (2)	C17—C15—C16	118.51 (17)
C10—C1—C11	118.0 (2)	C15—C16—C11	112.38 (17)
C2—C1—C11	123.5 (2)	C15—C16—C11A	112.5 (5)
C10—C1—C11A	141.8 (6)	C15—C16—H16A	109.1
C2—C1—C11A	98.6 (7)	C11—C16—H16A	109.1
C3—C2—C1	121.2 (2)	C11A—C16—H16A	130.3
C3—C2—H2	119.4	C15—C16—H16B	109.1
C1—C2—H2	119.4	C11—C16—H16B	109.1
C2—C3—C4	120.6 (2)	C11A—C16—H16B	83.0
C2—C3—H3	119.7	H16A—C16—H16B	107.9
C4—C3—H3	119.7	C18—C17—C26	117.78 (19)
C3—C4—C9	118.6 (2)	C18—C17—C15	121.89 (18)
C3—C4—C5	122.3 (2)	C26—C17—C15	120.30 (18)
C9—C4—C5	119.2 (2)	C17—C18—C19	122.21 (19)
C6—C5—C4	120.0 (2)	C17—C18—H18	118.9
C6—C5—H5	120.0	C19—C18—H18	118.9
C4—C5—H5	120.0	C20—C19—C18	122.3 (2)
C5—C6—O4	125.8 (2)	C20—C19—C24	118.9 (2)
C5—C6—C7	120.7 (2)	C18—C19—C24	118.82 (19)
O4—C6—C7	113.5 (2)	C21—C20—C19	120.6 (2)
C8—C7—C6	120.8 (2)	C21—C20—H20	119.7
C8—C7—H7	119.6	C19—C20—H20	119.7
C6—C7—H7	119.6	C20—C21—C22	120.3 (2)
C7—C8—C9	120.4 (2)	C20—C21—H21	119.9
C7—C8—H8	119.8	C22—C21—H21	119.9
C9—C8—H8	119.8	C23—C22—C21	120.7 (2)
C8—C9—C10	121.9 (2)	C23—C22—H22	119.7
C8—C9—C4	118.9 (2)	C21—C22—H22	119.7
C10—C9—C4	119.2 (2)	C22—C23—C24	120.6 (2)
C1—C10—C9	121.9 (2)	C22—C23—H23	119.7
C1—C10—H10	119.0	C24—C23—H23	119.7
C9—C10—H10	119.0	C25—C24—C23	122.6 (2)
C1—C11—C12	111.9 (2)	C25—C24—C19	118.46 (19)
C1—C11—C16	112.00 (18)	C23—C24—C19	119.0 (2)
C12—C11—C16	109.0 (2)	C26—C25—C24	121.5 (2)
C1—C11—H11	107.9	C26—C25—H25	119.3
C12—C11—H11	107.9	C24—C25—H25	119.3
C16—C11—H11	107.9	C25—C26—C17	121.3 (2)
C12A—C11A—C16	109.2 (12)	C25—C26—H26	119.4
C12A—C11A—C1	102.1 (11)	C17—C26—H26	119.4
C16—C11A—C1	103.6 (9)	O2—C27—O3	125.1 (2)
C12A—C11A—H11A	113.6	O2—C27—C12	126.6 (2)
C16—C11A—H11A	113.6	O3—C27—C12	108.4 (2)
C1—C11A—H11A	113.6	O2—C27—C12A	91.6 (6)
C27—C12—C13	107.72 (19)	O3—C27—C12A	143.1 (6)
C27—C12—C11	111.5 (2)	O3—C28—C29	109.9 (2)
C13—C12—C11	110.33 (19)	O3—C28—H28A	109.7
C27—C12—H12	109.1	C29—C28—H28A	109.7
C13—C12—H12	109.1	O3—C28—H28B	109.7
C11—C12—H12	109.1	C29—C28—H28B	109.7
C11A—C12A—C27	105.1 (11)	H28A—C28—H28B	108.2
C11A—C12A—C13	97.3 (10)	C28—C29—H29A	109.5
C27—C12A—C13	99.4 (8)	C28—C29—H29B	109.5
C11A—C12A—H12A	117.3	H29A—C29—H29B	109.5
C27—C12A—H12A	117.3	C28—C29—H29C	109.5
C13—C12A—H12A	117.3	H29A—C29—H29C	109.5
O1—C13—C14	122.4 (2)	H29B—C29—H29C	109.5
O1—C13—C12	120.3 (2)	O4—C30—H30A	109.5
C14—C13—C12	117.33 (19)	O4—C30—H30B	109.5
O1—C13—C12A	118.3 (5)	H30A—C30—H30B	109.5
C14—C13—C12A	109.8 (5)	O4—C30—H30C	109.5
C15—C14—C13	123.56 (19)	H30A—C30—H30C	109.5
C15—C14—H14	118.2	H30B—C30—H30C	109.5
C13—C14—H14	118.2		
			
C10—C1—C2—C3	−0.2 (3)	O1—C13—C14—C15	174.5 (2)
C11—C1—C2—C3	−178.5 (2)	C12—C13—C14—C15	−7.7 (3)
C11A—C1—C2—C3	170.0 (5)	C12A—C13—C14—C15	28.9 (7)
C1—C2—C3—C4	−0.7 (4)	C13—C14—C15—C17	−175.25 (19)
C2—C3—C4—C9	1.4 (3)	C13—C14—C15—C16	3.5 (3)
C2—C3—C4—C5	−178.6 (2)	C14—C15—C16—C11	−27.2 (3)
C3—C4—C5—C6	−179.7 (2)	C17—C15—C16—C11	151.59 (19)
C9—C4—C5—C6	0.3 (3)	C14—C15—C16—C11A	3.8 (8)
C4—C5—C6—O4	179.8 (2)	C17—C15—C16—C11A	−177.4 (8)
C4—C5—C6—C7	0.9 (4)	C1—C11—C16—C15	178.07 (19)
C30—O4—C6—C5	4.9 (4)	C12—C11—C16—C15	53.7 (3)
C30—O4—C6—C7	−176.1 (2)	C1—C11—C16—C11A	81.8 (11)
C5—C6—C7—C8	−0.6 (4)	C12—C11—C16—C11A	−42.5 (11)
O4—C6—C7—C8	−179.6 (2)	C12A—C11A—C16—C15	−46.9 (13)
C6—C7—C8—C9	−1.1 (4)	C1—C11A—C16—C15	−155.1 (6)
C7—C8—C9—C10	−178.4 (2)	C12A—C11A—C16—C11	48.9 (11)
C7—C8—C9—C4	2.3 (3)	C1—C11A—C16—C11	−59.3 (10)
C3—C4—C9—C8	178.2 (2)	C14—C15—C17—C18	−167.49 (19)
C5—C4—C9—C8	−1.9 (3)	C16—C15—C17—C18	13.7 (3)
C3—C4—C9—C10	−1.2 (3)	C14—C15—C17—C26	14.7 (3)
C5—C4—C9—C10	178.7 (2)	C16—C15—C17—C26	−164.12 (18)
C2—C1—C10—C9	0.3 (3)	C26—C17—C18—C19	−0.3 (3)
C11—C1—C10—C9	178.7 (2)	C15—C17—C18—C19	−178.19 (18)
C11A—C1—C10—C9	−164.0 (9)	C17—C18—C19—C20	−179.7 (2)
C8—C9—C10—C1	−179.0 (2)	C17—C18—C19—C24	0.3 (3)
C4—C9—C10—C1	0.4 (3)	C18—C19—C20—C21	−179.9 (2)
C10—C1—C11—C12	−155.9 (2)	C24—C19—C20—C21	0.1 (3)
C2—C1—C11—C12	22.4 (3)	C19—C20—C21—C22	−0.3 (4)
C11A—C1—C11—C12	47.9 (11)	C20—C21—C22—C23	0.0 (4)
C10—C1—C11—C16	81.3 (3)	C21—C22—C23—C24	0.4 (4)
C2—C1—C11—C16	−100.3 (3)	C22—C23—C24—C25	179.7 (2)
C11A—C1—C11—C16	−74.8 (11)	C22—C23—C24—C19	−0.6 (3)
C10—C1—C11A—C12A	−83.0 (12)	C20—C19—C24—C25	−180.0 (2)
C2—C1—C11A—C12A	110.9 (10)	C18—C19—C24—C25	0.0 (3)
C11—C1—C11A—C12A	−47.8 (10)	C20—C19—C24—C23	0.3 (3)
C10—C1—C11A—C16	30.4 (15)	C18—C19—C24—C23	−179.72 (19)
C2—C1—C11A—C16	−135.7 (8)	C23—C24—C25—C26	179.3 (2)
C11—C1—C11A—C16	65.6 (11)	C19—C24—C25—C26	−0.4 (3)
C1—C11—C12—C27	58.9 (3)	C24—C25—C26—C17	0.5 (3)
C16—C11—C12—C27	−176.69 (19)	C18—C17—C26—C25	−0.1 (3)
C1—C11—C12—C13	178.5 (2)	C15—C17—C26—C25	177.8 (2)
C16—C11—C12—C13	−57.1 (3)	C28—O3—C27—O2	−1.0 (4)
C16—C11A—C12A—C27	175.2 (9)	C28—O3—C27—C12	179.3 (2)
C1—C11A—C12A—C27	−75.6 (11)	C28—O3—C27—C12A	−173.7 (9)
C16—C11A—C12A—C13	73.3 (12)	C13—C12—C27—O2	−81.2 (3)
C1—C11A—C12A—C13	−177.5 (8)	C11—C12—C27—O2	40.0 (3)
C27—C12—C13—O1	−25.2 (3)	C13—C12—C27—O3	98.5 (2)
C11—C12—C13—O1	−147.1 (2)	C11—C12—C27—O3	−140.3 (2)
C27—C12—C13—C14	156.9 (2)	C13—C12—C27—C12A	−74.2 (9)
C11—C12—C13—C14	35.1 (3)	C11—C12—C27—C12A	46.9 (9)
C27—C12—C13—C12A	71.6 (9)	C11A—C12A—C27—O2	133.7 (10)
C11—C12—C13—C12A	−50.3 (9)	C13—C12A—C27—O2	−126.0 (7)
C11A—C12A—C13—O1	148.1 (8)	C11A—C12A—C27—O3	−52.2 (15)
C27—C12A—C13—O1	41.3 (10)	C13—C12A—C27—O3	48.1 (13)
C11A—C12A—C13—C14	−64.7 (10)	C11A—C12A—C27—C12	−40.7 (9)
C27—C12A—C13—C14	−171.5 (5)	C13—C12A—C27—C12	59.6 (7)
C11A—C12A—C13—C12	45.0 (9)	C27—O3—C28—C29	−100.8 (3)
C27—C12A—C13—C12	−61.8 (8)		
